# ‘Quit During COVID-19’—staying smokefree in mental health in-patient settings

**DOI:** 10.3332/ecancer.2020.ed102

**Published:** 2020-06-04

**Authors:** Pooja Patwardhan, Richard Driscoll

**Affiliations:** 1 Centre for Health Research and Education, UK; 2 Visiting Consultant, Bristol Priory Hospital, UK and Centre for Health Research and Education, UK

**Keywords:** mental health, smoking cessation, COVID-19, tobacco harm reduction, e-cigarette, NRT, smokefree mental health, smoking

## Abstract

Cigarette smoking is one of the main preventable causes of cancers globally. At this time of global emergency, mental health professionals all over the world are joining hands with the public health and other healthcare communities to focus on acute measures to save lives from COVID-19. This has been particularly challenging in mental health hospital settings where numerous additional factors need to be considered, including difficulties of implementing social distancing, potential impacts of social isolation, increased stress levels and implications of all this on smoking. In this article, we will briefly discuss the prevalence of smoking in mental health patients, especially in those in mental health hospital settings and also what is the possible impact of COVID-19 pandemic in these people. Then we will go through the main reasons as to why encouraging smoking cessation in mental health patients is so important and measures we can take for supporting mental health patients quit smoking even during COVID-19 times. The smoking cessation interventions have a direct bearing on preventing future cancers and achieving smoking cessation among cancer patients in this already disadvantaged group.

## Smoking in mental health patients and smokefree site policies in pre-COVID times

Smoking rates among people with a mental health condition are disproportionately higher than average national smoking rates across the world [1–3]. The association between smoking and mental health conditions becomes stronger relative to the severity of the mental health condition, with the highest levels of smoking found in psychiatric in-patients [2–4]. People with mental health conditions die on average 10-20 years earlier than the general population, and smoking is the single largest factor accounting for this difference [3]. We also already know that smoking is the largest preventable cause for multiple types of cancers in the world.

Mental health hospitals in many countries such as the UK have a length of inpatient stay of weeks to months. The patient turn-over is not as quick as in acute hospital settings. Patients often need to live there as if they are staying at their homes but with support from hospital staff to benefit from the treatments. Many patients normally are allowed to go out of the hospital premises on a regular basis on escorted and unescorted leaves, for rehabilitation activities and to socialise in the community, as well as within the hospital grounds. In pre-COVID-lockdown times, smoking opportunities still existed for smokers on/near mental health sites or during their unescorted leave, although it was considered poor practice to support smoking on periods of escorted leave. With this background, the NHS (the National Health Service), as well as private mental health hospital sites in the UK were asked to go smokefree over the last few years [5]. The experience and partially successful attempts of NHS mental health trusts in going smoke-free between 2015 and 2018 are well documented [6,7].

In August 2019, the Centre for Health Research and Education (CHRE) conducted a survey among a convenience sample of 325 staff across mental health sites in the UK (unpublished research). The UK is known to be a world leader in smoking cessation knowledge and policies; and yet 52.3% of mental health site staff surveyed believed that ‘nicotine in cigarettes causes cancer’. So even before the COVID-19 pandemic started, the knowledge about nicotine amongst mental health staff was very poor. This finding is previously reported from the mental health professionals’ community but is not unique to them [8]. Studies in the past among GPs and in the general population have given similar results [9,10]. In mental health settings, however, the impact of this lack of understanding of nicotine in staff could be a key contributing factor to poor cessation outcomes in mental health inpatients, and this further demoralises staff in championing smokefree policies [11].

## Effects of COVID-19 on mental health of the population and related challenges in mental health hospitals

The COVID-19 pandemic, apart from the effects on physical health due to serious respiratory infection and complications, is also causing serious effects on the general population’s mental health and general mood [12,13]. With the world going into lockdown, social distancing and self-isolation are likely to make the society very lonely and life more stressful. Chances of increased anxiety or even fear as a result of health and financial uncertainties are high. It has been predicted that symptoms of depression may be displayed across all age groups as a result of social isolation [14–18]. Similar stress levels and reactions were seen in respiratory epidemics needing isolation, like SARS and Middle East respiratory syndrome, in the past [17–19].

In addition to the above reasons for worsening mental health of people in the general population, in mental health services in-patients are characteristically often acutely psychotic and as such may be experiencing delusions, hallucinations, disordered thinking, disinhibition, dysregulation of affect and behaviour and impairment of decision making capacity. Consequently, the ward milieu is fragile, the patients are vulnerable and there are multiple challenges to patient and ward management around implementing leave restriction and social distancing in mental health hospitals, during the COVID-19 pandemic. Keeping 2m from others on the wards and providing a positive environment for mental wellbeing while allowing mental health patients to go out only once each day for exercise or other health care appointments can be significantly challenging in in-patient settings and can increase mental stress for patients as well as staff. Previously available opportunities to smoke on/near site [11] or when on leave are not available during COVID-19 lockdown, thus disturbing the permissive culture and arrangement between staff and patients of off-site smoking. During COVID-19, some mental health hospital specific situations may be harder to manage, e.g., rapid tranquilisation and/or physically restraining agitated patients.

## Risk of relapsing and smoking more

Stress as well as boredom are known emotional triggers for smoking [17–22]. In the current unexpected and unprecedented times, there is a significant risk of increased smoking as a result of increased stress in the general population. We might also see increased relapse to smoking due to the sudden inaccessibility and unavailability of usual support mechanisms [23], with friends and family precluded from visiting, along with COVID-19 organisational measures significantly disrupting the normal running of the therapeutic ward activities and relationships that usually deliver support.

As mentioned before, smoking rates are significantly higher in patients with Severe Mental Illness (SMI) e.g. in patients diagnosed with schizophrenia, smoking prevalence can be as high as 80%. In mental health hospitals, we are starting to see that the smokefree policies are being relaxed as a quick-fix solution by the healthcare practitioners to help their patients cope in the short term and to handle the COVID-19 related challenges. Staff are often allowing smoker patients to smoke on site again. In this situation, patients as well as staff members who are smokers might smoke more than before, and ex-smokers might relapse back to smoking due to peer pressure and stress. However, it is now proven that smoking actually results in poorer mental health symptoms [8] and exacerbates stress [24]; and that stopping smoking is as effective as antidepressants [25] thus alleviating the symptoms of anxiety and depression.

## Smoking worsens physical and mental health outcomes

Mental health patients also are known to have higher incidence of obesity and hyperlipidaemia, either related to medication induced metabolic syndrome, the direct impact of mental ill health on behaviour, the consequences of institutional care or other inequalities [26]. This makes them already at a higher risk of COVID-19 complications. If they continue to smoke, the chances of complications are even more.

Smoking is known to increase the metabolism of many drugs, thus rendering them less efficacious and requiring higher dosing with their attendant adverse effects and risks. E.g. Clozapine, Haloperidol, Olanzapine, diazepam, etc. For many patients suffering from Treatment Resistant Schizophrenia, treatment with Clozapine is transformative. There is no current direct evidence that Clozapine is an independent risk factor for those infected with COVID-19 [27]. However, there is face validity to concerns that there may be an increased risk. Those on Clozapine already have higher rates of hospital admission for pneumonia which is both argued to be potentially due to reduced immunoglobulin levels and reported cases of aspiration pneumonia linked to Clozapine induced sialorrhea. Smoking significantly induces the metabolism of Clozapine, thus necessitating a dialling up of the dose of the drug and increasing the risk to patients of lung complications.

Healthcare professionals as well as smoker patients often overlook the risks due to smoking; not only the known long-term risks, but also those in the short term. The physical health impact of smoking is well documented and known. It significantly increases risk of not only heart and lung diseases, but also multiple types of cancers. Smoking is also proven to worsen the mental health treatment outcomes and prognosis, which is often forgotten by health care staff.

COVID-19 adds to the already known mental and physical health challenges associated with smoking in mental health patients. These are summarised in Table 1.

## Smokefree advice and support needs to continue during COVID-19

The lockdown around the world is leading to reduced access to face to face healthcare services including smokefree services. While the chances of increase in smoking are high, the motivation to quit has also slightly raised amongst some smokers to prevent COVID-19 complications [30]. Unfortunately, this is the worst time to leave smokers without enough cessation support as the chances of quitting smoking successfully without support are very low [31]. Providing bespoke stop smoking services to disadvantaged smokers, including those with mental health conditions, while the effects of COVID-19 pandemic are still there, can increase the likelihood of quitting success in the short to medium term [2,30]. If the governments, health care professionals and policy makers ignore the need for supporting smokers in these times of crisis, we might see another global health calamity due to smoking related morbidity and mortality after the COVID-19 pandemic is over [23]. The additional preventable loss of life to cancer and increased strain on the already stretched health systems will be tragic.

## Quit during COVID-19 in mental health: role of healthcare practitioners

Attempting and maintaining smoking cessation can dramatically enhance the chances of physical and mental health improvement in mental health patients. There is a particular undisputed need for mitigating against the consequences of lockdown and social isolation for vulnerable groups during the COVID-19 pandemic; and supporting and sustaining smoking cessation is key to meeting that need.

CHRE has been working with mental health sites across the UK to look into the hurdles and barriers to continuing to be smokefree and finding practical solutions for it, even before the COVID-19 pandemic. We have continued our work with the frontline mental health staff, using innovative ways during the COVID-19 pandemic, which is unknowingly being overlooked by many.

Our practice-oriented advisory to all the healthcare practitioners for supporting smoker patients quitting smoking during COVID-19 is briefly summarized below:

- Discuss the risk of **increased likelihood for COVID-19 complications** with patients and with your team members. This might be the best time to quit smoking permanently!
- Health care staff need to be clear about the **effects of Nicotine** and that it is not carcinogenic. They also need to practice the evidence-based **principle of harm reduction to help smokers quit smoking** by offering safer nicotine products like NRT and e-cigarettes (in countries where they are legal and regulated).
- ***Encourage all smokers cut down and quit smoking.*** Advise them to use enough safer nicotine to stay away from smoking. Most smokers need to use ***nicotine patches with short acting NRT and/ or e-cigarettes as a ‘dual therapy’***.
- ***Educate patients on managing acute cravings*** with adequate and timely nicotine replacement using ***short acting NRT and/ or e-cigarettes**** (in countries where they are legal and regulated).*
- Consider medications such as ***Varenicline*** [32] in the mental health hospital setting. It is safe and a very effective cessation aid in most mental health patients.
- ***Provide behavioural support*** through ***behavioural therapy apps/ online tools and advice on distraction techniques.***
- ***Discuss risk of relapse and how to avoid triggers with all patients who have managed to quit smoking.***

**Figure 1:**
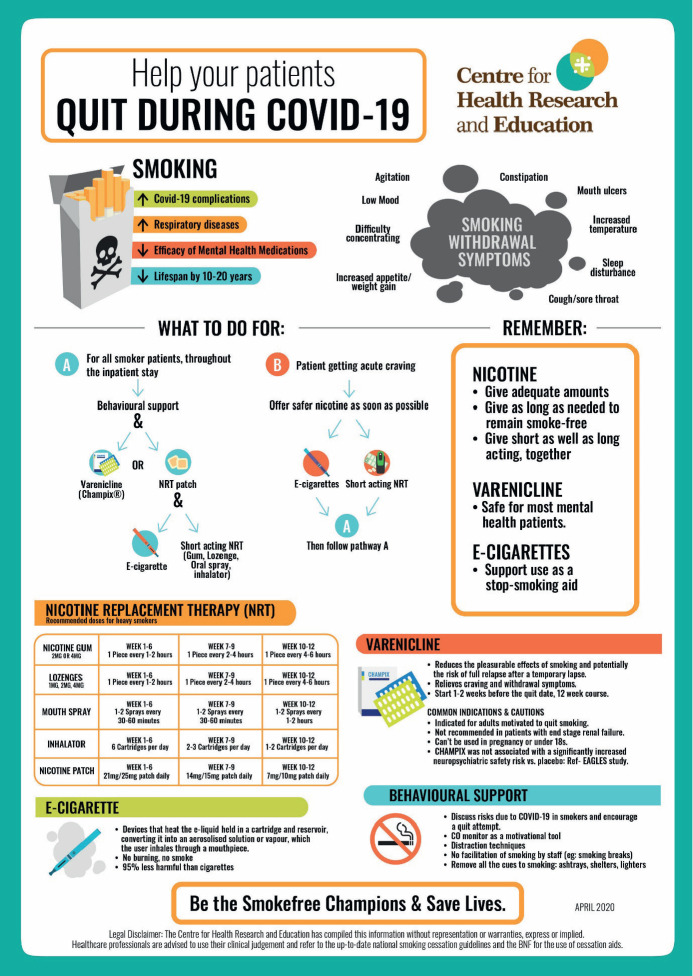
Quit during COVID-19 infographic.

Patients can get craving to smoke any time and it is possible that all the attending staff are not up to date with evidence-based latest guidance on smoking cessation treatments. If patients are given enough safer nicotine promptly and then followed by long term treatment options along with behavioural therapy, then the quitting success rates are likely to be higher. Figure 1 is a practice-oriented infographic created by CHRE for use by all the healthcare practitioners supporting smoker patients. It can be printed and put in clinics or wards for quick use or be referred to on their phones. This infographic can be adapted for use in any in-patient settings, including oncology wards and care homes, where supporting and maintaining smoking cessation can dramatically impact quality of life and patient outcomes. We are also piloting, for April and May 2020, a daily phone advice line staffed by a level 2 NCSCT trained stop smoking advisor. The advice line is for addressing any smokefree-mental health related queries and providing support to staff from mental health hospitals across the UK on the latest evidence-based approaches to smoking cessation.

## Conclusion

Going smokefree in mental health settings is already a challenge. Smoking worsens physical and mental health outcomes among mental health patients, and yet staff and patients struggle to be sustainably smokefree. Increased smoking due to COVID-19 threatens to increase the risk of ill health including higher cancer risk in already disadvantaged mental health patients. The gap between the smokefree policy and practice will change only if the frontline mental health staff are upskilled and empowered with practical tools and knowledge on safety of nicotine and tobacco harm reduction. We call for national public health bodies and all the health care professionals seeing mental health patients, to encourage and support all their smoker patients to ‘quit smoking during COVID-19’ and help them move towards a better positive and healthier life: during and after COVID-19!

## Conflicts of Interest

PP is a practising sessional GP in Hampshire, UK. PP is also a paid director for the Centre for Health Research and Education (CHRE) UK, an independent healthcare company. CHRE works on projects on smoking cessation globally. PP or CHRE have not received any funding from pharmaceutical, electronic cigarette or tobacco industries.

RD has received payments from the CHRE.

The contents, selection and presentation of facts, as well as any opinions expressed herein are the sole responsibility of the authors.

## Funding statement

CHRE has received a grant for a project in Smokefree Mental Health from The Foundation for Smokefree World.

## Figures and Tables

**Table 1:** smoking health risks and its implications to Mental Health patients

Smoking : health risks in MH patients	Implications
Increases risk of other respiratory infections [26] and exacerbates conditions such as asthma. Increase risk of serious complications from COVID-19 [28]	Risk to patients Increased burden on healthcare service
Reduces efficacy of many psychiatry medications [29]	Poor mental health symptom control Need for higher doses of medications giving more side effects Need for more blood tests for measuring drug levels e.g.: Clozapine
Contributing and worsening factor for mental illness [29]. Increased likelihood of smoking during COVID-19 due to lockdown and related stress.	Increased patient distress and burden on mental health staff
Increase long term risk of cardiovascular and respiratory diseases, and cancers [3].	Smoking is the single largest contributing factor for reducing the life span of those with severe mental illness by 10-20 years.
Increases risk of ex-smokers relapsing back to smoking due to the stress [23] and peer pressure in inpatient settings as a result of relaxed smokefree policies during COVID-19.	All the hard work put in to quit smoking reversed. Can take many years to get the culture to change back again to enable mental health hospitals to convey the positive health messages.
